# Side effects of COVID-19 vaccines in the middle eastern population

**DOI:** 10.3389/fimmu.2023.1270187

**Published:** 2023-11-03

**Authors:** Ghida M. Murished, Iman Dandachi, Waleed Aljabr

**Affiliations:** Research Center, King Fahad Medical City, Riyadh, Saudi Arabia

**Keywords:** COVID-19 vaccines, side effects, immune response, children, high-risk population

## Abstract

The COVID-19 pandemic has caused severe worldwide health concerns since its first description as the SARS-COV-2 virus in December 2019. The wide dissemination of this virus, together with the lack of treatment, prompted vaccine development within a short period of time to elicit a protective immunity against COVID-19. Due to their rapid development, potential subsequent side effects of COVID-19 vaccines were overlooked, which might lead to many health concerns. This is especially true for patients at a greater risk of harm from COVID-19, such as pregnant women, children, and patients with pre-existing chronic diseases. In this review, we provide a summary of common to rare side effects of administrated COVID-19 vaccines in a Middle Eastern population. We have found that the distinction between side effects from COVID-19 vaccines in terms of frequency and severity is attributed to the differences in study populations, gender, and age. Pain at the injection site, fever, headache, fatigue, and muscle pain were the most common reported side effects. Vaccinated subjects with previous COVID-19 infection exhibited an equivalent neutralizing response after just one dose compared to two doses of vaccine. Consequently, individuals who experienced more side effects had significantly higher antibody levels. This indicates that having better immunity correlates with higher antibody levels, leading to a higher frequency of vaccine side effects. Individuals with underlying comorbidities, particularly having known allergies and with illnesses such as diabetes and cancer, might be more prone to post-vaccination side effects. Studies of a high-risk population in Middle Eastern countries are limited. Future studies should be considered to determine long-term side effects, side effects after booster doses, and side effect differences in cases of heterologous and homologous vaccination for better understanding and proper handling of high-risk populations and patients who experience these side effects.

## Introduction

Severe acute respiratory syndrome coronavirus 2 (SARS-CoV-2), a member of the Coronaviridae family, is an enveloped, single-stranded, positive-sense RNA virus (1). The coronavirus family is characterized by its round/oval shape, with a crown-like appearance and a diameter of ~60–140 nm (2). Within this family, four genera exist: alpha, beta, delta, and gamma. Alpha and beta are generally responsible for infections in mammals and humans, while delta and gamma mainly infect birds (2). Following its first detection in late December 2019, in China (3), SARS-COV-2 has rapidly spread in many countries, causing millions of deaths; accordingly, the World Health Organization (WHO) declared COVID-19 a worldwide pandemic on 11^th^ of March 2020 (4). The dissemination of SARS-COV-2 did not only impact human health, but also affected the mental and physical behavior of individuals (5).

The prognosis of COVID-19 ranges from an asymptomatic infection to a severe life-threatening disease (6). Unfortunately, treatment of COVID-19 is only supportive, with no specific antivirals available yet (7). Generating immunity to handle its dissemination through vaccination was therefore the main approach to combat it (7). Countries all over the globe were in a rush to develop effective vaccines to protect their populations. In less than a year, by December 2020, many SARS-CoV-2 vaccines were approved for emergency use in different parts of the world, including chimpanzee adenovirus vector vaccines (ChAdOx1-Astrazeneca), spike-gene RNA-based vaccines, (BNT162b2 Pfizer–BioNTech), and human adenovirus 26 (Ad26.COV2.S-Johnson & Johnson/Janssen) (8). Middle eastern countries were among the first to start COVID-19 vaccination campaigns; the Kingdom of Saudi Arabia (KSA) and Bahrain started in the middle of December 2020, while Israel started their campaign two days later (9–11). Qatar, Kuwait, Oman (12, 13), Jordan, the United Arab Emirates (UAE) (9, 14, 15), and Egypt (16) began their vaccination programs in January 2021.(ElSharkawy, 2021). In February 2021 Iran (17), Lebanon (18), and Palestine (19) began vaccinating; Yemen began in March 2021 (20), and in May 2021 Iraq (21) and Syria (22) started their vaccination programs. Priority for vaccination was at first given to people who were at an increased risk of contracting the SARS-COV-2 infection, including frontline health care workers (HCW), immunocompromised individuals, and the elderly (23). Thereafter, SARS-COV-2 vaccines became readily administered to all individuals (Centers for Disease Control and Prevention, 2022), including pregnant women and children aged between 12 and 18 years (24). As of October 2022, the number of administered doses of COVID-19 vaccines in all middle eastern countries exceeded 20 million (4).

In vaccine development, the vaccine must undergo six primary stages: preclinical, clinical phase 1-3, approval, and and manufacturing and post-marketing surveillance (25). In the case of COVID-19, in view of the pressing need, the development process was sped up, with some phases being merged. As a result, COVID-19 vaccines were produced earlier than planned, together with an increased risk of potential vaccine-related side effects (26) (27). According to the Centers for Disease Control and Prevention (CDC), common side effects include pain, redness, or swelling at the injection site, fever, muscle and joint aches, headaches, and chills (28). Rare/serious side effects include Guillain-Barr´e syndrome (GBS) (29), extensive deep vein thrombosis, pulmonary thromboembolism (30), nephrotic syndrome, acute kidney injuries (31), and vesiculobullous skin reactions (32). However, the most commonly reported side effects were the minor ones and indicated the development of immunity. Surveillance of the prevalence of other side effects is still essential, though, to predict the overall consequences of the SARS-COV-2 vaccines. This is especially true for the vaccines developed with new technology that involves mRNA technology, such as Pfizer-BioNTech and Moderna vaccines (33, 34). Moreover, assessing the duration of side effects after each dose (35, 36) as well as connecting their frequency, severity, and duration with possible factors such as previous COVID-19 infection, age, sex, and other immunocompromising conditions will help for better understanding of these adverse events. The aim of this review is to summarize the current available data on the SARS-COV-2 vaccines reported side effects, as well as exploring if there is a relation between these adverse events with specific demographical and/or clinical characteristics in the middle eastern population. The middle eastern countries include the gulf region: KSA, UAE, Qatar, Kuwait, Bahrain, Oman, Yemen, the levant region including Lebanon, Jordan, Palestine, Syria, Egypt, and Iran.

## Search strategy and data sources

**Figure 1:**
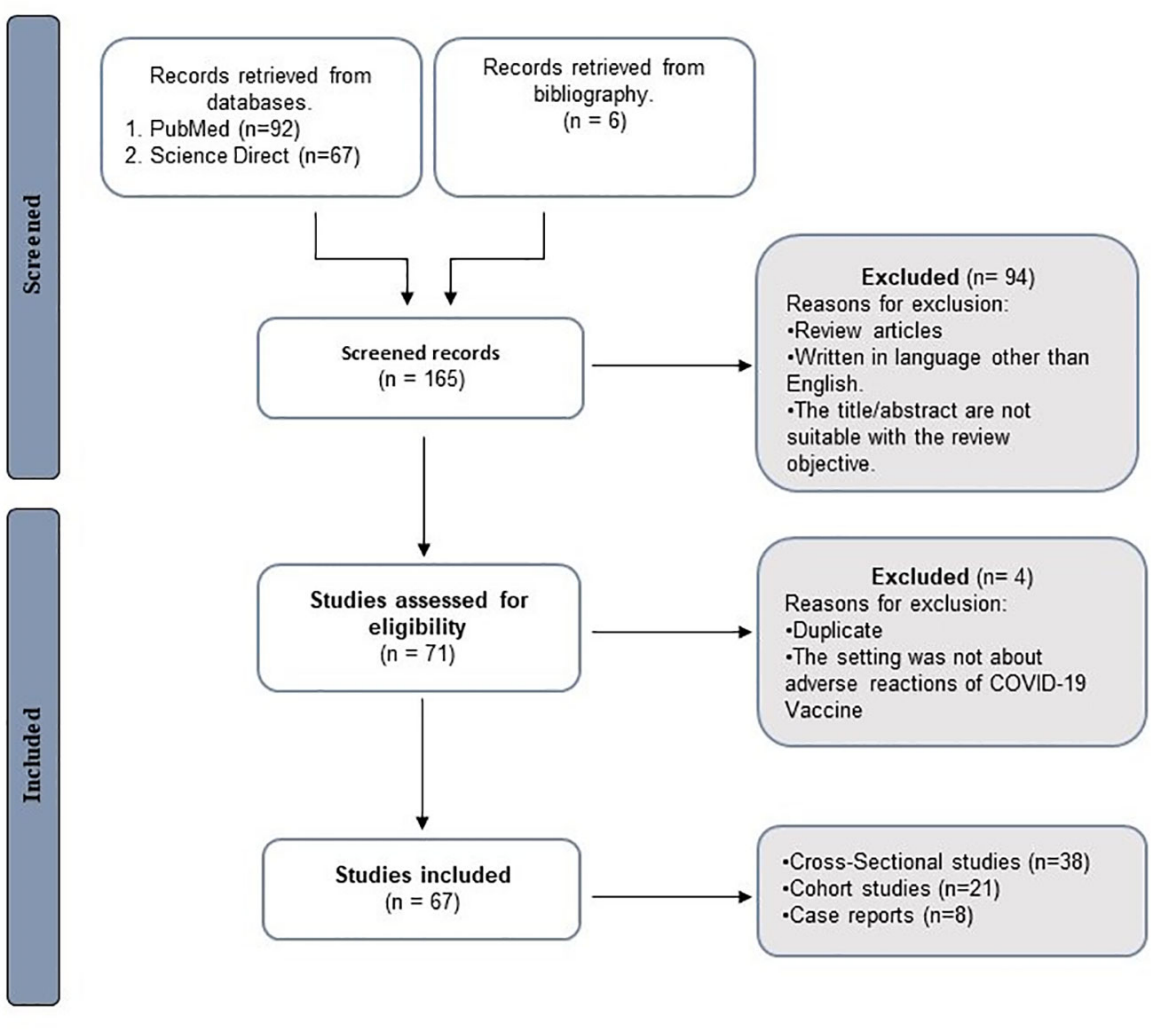
Review design flow chart. N refers to number.

PubMed and Science direct were searched for all papers discussing the side effects of COVID-19 vaccines in the middle eastern population, from 2020 to March 2023. The following keywords were used: “COVID-19”, “SARS-CoV-2”, “adverse effects”, “side effects”, “vaccination”, “immune response”, “complications”, “middle eastern countries”, “comorbidities”, “Pfizer”, “Astrazeneca”, “Johnson & Johnson”, “Moderna”, “Sinovac”, “Sinopharm”, “Sputnik”, “Covaxin”, “children”, and “high-risk population”. Papers were included if the language of writing was English and if the results provide clear information of the COVID-19 vaccines’ side effects. On the other hand, papers were excluded if they were duplicates, no clear information was provided, or if they were written in non-English language. The search and inclusion strategy is shown in **Figure 1**.

## Health care workers

In all countries, health care workers are considered as the most valuable resource in the health care system. During the COVID-19 pandemic, HCW were at the frontlines to combat the disease, which made them at a higher risk of contracting the infection, due to their frequent exposure to the virus (37). In addition, HCW experienced physical and mental exhaustion due to having to make difficult decisions, the dramatically increased number of infected patients, and death including their colleagues. When vaccines were implemented, the global response was to first target this population and to ensure its safety (38).

In Saudi Arabia, Ahsan et al., found that fever higher than 39°C, dyspnea, and anxiety were the main side effects experienced by HCW after Astrazeneca and Pfizer BioNTech vaccinations. Other less commonly observed side effects were low-grade fever, chills, headache, and pain at the injection site, followed by dyspnea and anxiety (39) (**Table 1**). It is worth mentioning that subjects who experienced the major side effects were more likely to have drug allergies such as to penicillin, as well as food and other allergies such as soya, nuts, and dust (39). This finding might indicate an interrelation between allergies and post-vaccination side effects. In another study, it has been found that for mRNA-based COVID-19 vaccines, tiredness was the most common side effect, followed by pain at the injection site, fever, myalgia, and headache (40). Compared to Astrazeneca, the frequency of side effects was higher in Pfizer administered to HCW (40). Moreover, it has been shown that female HCW had more severe side effects compared to male ones after both Pfizer BioNTech and Oxford-Astrazeneca vaccines (39, 40). In addition, it was found that the severity and frequency of side effects decreased with increasing age; younger people aged between 27-36 years old reported more major side effects compared to older ones (39). These findings are in accordance with reports from the United States and Italy, where it was shown that younger individuals and female HCWs tended to experience more side effects after vaccination (60, 61).

**Table 1:** COVID-19 Vaccine side effects in HCWs, high-risk groups, and children in the Middle East.

Country	Vaccine type	Administered doses	Studied population	Major common side effects reported	Minor less common side effects reported	Rare side effects	Reference
**KSA**	Pfizer, AstraZeneca	At least 1 dose	HCW	High grade fever, dyspnea, anxiety	H/F, chills, PIS, myalgia, arthralgia, fatigue		(39)
	mRNA-based vaccine	1 or 2 doses	HCW		Tiredness, PIS, H/F, myalgia		(40)
**Israel**	Pfizer	2 doses	HCW	Redness, swelling, PIS			(41)
**Iran**	Sputnik V	1 or 2 doses	HCW	PIS, fatigue, body/joint pain, H/F, chills	Diarrhea, depression	Increased heart rate, itching, SOB, dry/bad mouth, temporary hair loss, runny nose, sore throat	(42)
	Sputnik V, Sinopharm, Astra, Covaxin, Barekat	1 or 2 doses	HCW	Fatigue, body/joint pain, H/F, chills, tenderness & PIS	Rash, vomiting, constipation, anaphylaxis shock, vasovagal syncope		(43)
	Sputnik V, Astra, Covaxin	first dose	HCW	PIS, muscle pain, fatigue, fever, chills	Swollen lymph nodes		(44)
	Sputnik V, Sinopharm, Astra, Covaxin	1 or 2 doses	HCW	PIS, body/muscle pain, H/F, fatigue			(45)
**Iraq**	Pfizer, Astra, Sinopharm		HCW	PIS, H/F, myalgia, tiredness	Insomnia, lymphadenopathy, somnolence, metallic taste, joint/back pain		(46)
	Pfizer, Astra, Sinopharm	1 or 2 doses	HCW	PIS, MJP, H/F, fatigue, feeling sick/achy, redness/pruritus at injection site, cough, nausea, LOS & taste, diarrhea			(47)
**Jordan**	Pfizer, Astra, Sinopharm	1 or 2 doses	HCW	Numbness & PIS, fatigue, myalgia, H/F, arthralgia, bone pain.	Cough, diuresis, HZV, sleepiness, loin/chest pain, CC, LOS, LBP, palpitations, R/S at injection site, thirst, urticaria		(14)
**Israel**	Pfizer	1 or 2 doses	High Allergy	Fatigue, headache, muscle pain		Skin eruption, tongue/uvula swelling, cough, bronchospasm, itching, SOB, angioedema, GI	(48)
	Pfizer	1 or 2 doses	AIRD			Non-disseminated HZV, uveitis, pericarditis	(49)
**Oman**	Astra		SCD			Hb drop, raise in liver enzymes, severe VOC, fatal TTP-like syndrome, thromboembolic complications	(50)
	Sputnik V, Sinopharm, Astra, Pfizer	1 or 2 doses	Multiple sclerosis	Injection site reaction, new rash onset, flare-up of pre-existing dermatological conditions, urticaria, generalized pruritus, maculopapular rash			(51)
**Iran**	Sinopharm	First dose	Multiple sclerosis	Malaise, H/F, shivering, body pain,	GI discomfort, respiratory symptoms	Multiple sclerosis relapse	(52)
	Sinopharm	1 or 2 doses	Multiple sclerosis			Multiple sclerosis relapse, COVID-19 contraction, ulcerative colitis flare	(53)
	Sinopharm, Astra	1 or 2 doses	Multiple sclerosis	Redness & PIS, tenderness, H/F, fatigue, nausea, diarrhea, muscle pain			(54)
**KSA**	Pfizer	1 or 2 doses	Children 12-18 y	Redness & PIS, fatigue, H/F, N/V, chest pain, SOB	Joint/bone pain		(55)
	Pfizer	1 or 2 doses	Children	R/S & PIS, H/F, tiredness, MJP, N/V, malaise, chest stiffness	Change in mouth odor, swollen lymph nodes, mouth ulcers/numbness, itching, skin rash, lips corner infection, gum bleeding,		(56)
**Iran**	Sinopharm, Sobera	1 or 2 doses	Children & Adolescents 10-18 y	Fatigue, fever, chills, PIS, dizziness		Vascular injuries, respiratory complication	(57)
**Israel**	Pfizer	2 doses	Adolescents 16–18 y			Transient cardiac injury	(58)
	Pfizer	2 doses	Children & Adolescents 12-15 y			Myocarditis	(59)

SCD, Sickle Cell Disease; y, years old; Astra, AstraZeneca; SOB, shortness of breath; GI, gastrointestinal symptoms; HZV, herpes zoster; PIS, pain at injection site; AIRD, Autoimmune inflammatory rheumatic disease; VOC, vascular-occlusive crisis; LOS, loss of smell; LBP, Lower back pain; CC, common cold; H/F, Headache/fever; N/V, nausea/vomiting; Hb, hemoglobin; R/S, redness and swelling; MJP, muscle & joint pain.

In Israel, more systemic adverse reactions were observed following the second dose of the Pfizer BioNTech vaccine compared to the first dose, with local or no side effects (41). Interestingly, it has been found that subjects who had more side effects, including pain, redness, and swelling at the injection site, or any other systemic side effects, had a notably higher level of antibodies than those who did not experience as many as side effects. This indicates a possible correlation between having better immunity i.e., a higher antibodies levels, and experiencing more side effects (41).

In Iran, the most common side effect reported after vaccination with Sputnik V, Covaxin, Astrazeneca (42–44), and COVIran Barekat (43) was pain at the injection site. Fatigue, muscle pain, and fever were more commonly reported following the first dose of Sputnik V compared to the second dose in one study (42). Other studies have shown that the frequency of these side effects was equally reported for Covaxin, Astrazeneca, Sputnik V, and COVIran Barekat vaccines (43, 44). On the other hand, the prevalence and frequency of other common side effects such as body pain, headache, joint pain, chills, and drowsiness were reported differently (42, 44), with these being significantly less prominent following the second dose of Sputnik V, AZD-1222, and Covaxin compared to the first dose (44). For Sputnik V specifically, studies in Iran have shown that less observed side effects after the first and second dose included diarrhea, vomiting, constipation, allergic reactions, vasovagal syncope, depression, rash, abdominal pain, blurred vision, dyspnea, pruritus, palpitations, malaise, and swollen lymph nodes (42, 43). Some rarely reported side effects for this same vaccine includes increased heart rate, hair loss, shortness of breath, itching all over the body, bad taste, dry mouth, runny nose, sore throat, and seizure (42) (43). Females and younger individuals, aged 38-40 years old, are more likely to experience Sputnik V side effects (42, 44, 45), such as injection site pain, fatigue, headache, or fever (44). Women with comorbidities, specifically a history of anaphylaxis to previous vaccines or multiple drugs, experienced more of these side effects than those without comorbidities (45). Reports from Argentinian and Czechian healthcare workers reported that pain at the injection site and muscle pain were the most frequently observed local and systemic side effects, after Sputnik V, Pfizer-BioNTech, as well as the Oxford-Astrazeneca vaccines (62, 63). Similar side effects such as fatigue, body pain, and headache were also reported in studies from Pagotto et al. and Riad et al. (62, 63). The same side effects were also reported among HCWs in Indonesia after the Sinovac vaccine (64). Taken together, these findings reveal that side effects such as fatigue, pain at the site of injection, headache, and fever were commonly reported in several studies that addressed different vaccines platforms, implying that these effects are expected in all HCW after taking the COVID-19 vaccine. As for the effect of previous infection with SARS-COV-2 on the side effects after vaccination, it has been found in Iran that individuals with a history of COVID-19 infection experienced a significantly higher rate of side effects, such as injection site and muscle pain, fatigue, weakness, and body pain (42, 44). The incidence rate of these side effects in convalescent patients who received Sputnik V and Covaxin vaccines was higher (44). Moreover, individuals who had previously contracted COVID-19 reported lower incidence of side effects such as joint pain, fever, and headache, compared to those without prior infection, before vaccination (42).

In Iraq, only one study exploring the side effects observed following vaccination in HCW was conducted. In this, it has been found that moderate to severe pain in the injection site was the most frequently observed side effect after Pfizer and Astrazeneca vaccines. Compared to Sinopharm, Pfizer and Astrazeneca vaccines had higher frequencies of side effects including tiredness, headache, myalgia, fever, and chills, with most of these being reported after the first dose. Furthermore, Pfizer-BioNTech and Astrazeneca were the only vaccines where rare side effects such as mild to moderate palpitation and gastro-intestinal symptoms were observed, as well as diarrhea Some of the HCWs experiencing these uncommon effects consulted a physician, in part because they themselves are medical staff (46).

The correlation of Astrazeneca and Pfizer BioNTech vaccines with a higher frequency of side effects, compared to Sinopharm, was also shown in a study conducted in Jordan. Specifically, Astrazeneca was significantly associated with more severe side effects (14). Pfizer BioNTech was associated with more local side effects, and SinoPharm was not associated with any side effects, indicating its weak immunogenic potential (14). Fatigue was the most common side effect reported after both doses of these vaccines, followed by pain in the injection site, numbness, headache, fever, myalgia, arthralgia, as well as bone pain (14). The severity of these mentioned side effects was observed at a lower rate after the second dose; this is probably due to the difference in numbers of vaccine recipients between the first and second doses. The Astrazeneca vaccine, which was found to be highly associated with side effects in their study, was given to fewer than 4% of second dose recipients (compared with 44% of first dose recipients). In contrast, approximately 45% of second dose recipients received SP vaccines, which have minimal side effects (14). Individuals aged 45 years old or younger developed more systemic side effects, especially fatigue, myalgia, headache, and fever (14).

## General populations

**Figure 2:**
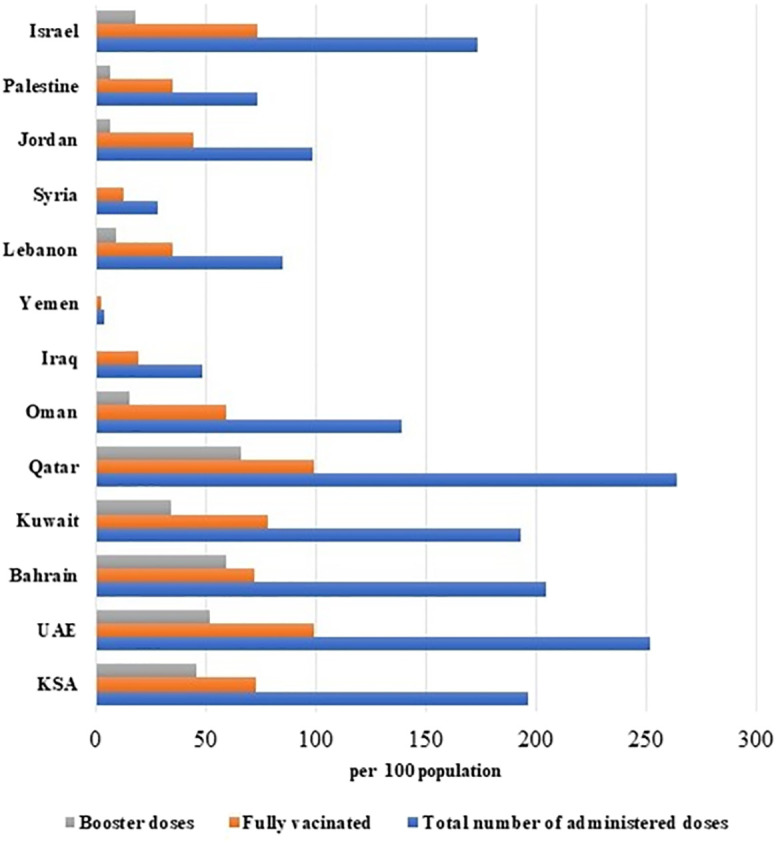
Vaccination status in middle eastern countries.

As mentioned earlier, COVID-19 vaccination campaigns in middle eastern countries started between December 2020 and January 2021. Vaccination of the general population started immediately after high-risk groups. By the end of October 2022, the number of administered doses exceeded 20 million across all middle eastern countries (**Figure 2**).

In Oman, Ghafri et al, found that after the receipt of the second dose of Astrazeneca, individuals experienced fever, chills, headache, body ache, malaise, and fatigue. Subjects who took Pfizer BioNTech, meanwhile, had an allergic reaction within 24 hours of administration and required medical attention (12). Astrazeneca-linked side effects were reported more frequently than Pfizer BioNTech-linked side effects, possibly due to differences in their nature of action; Astrazeneca utilizes a live-attenuated approach while Pfizer BioNTech is an mRNA-engineered vaccine (12). For both vaccines, side effects were more commonly reported in women, younger people, and those with a history of allergy and asthma (12). Cutaneous side effects have been also reported following vaccination with Pfizer-BioNTech, Astrazeneca, and Sputnik (51). Flare-ups of pre-existing dermatological conditions including flare-ups of acne and psoriasis were reported as side effects after Pfizer-BioNTech, Astrazeneca, and Sputnik vaccines (51). This is in addition to lichen planus, pemphigus foliaceus, flare-ups of herpes simplex, and pompholyx (51). Other reported cutaneous side effects in the Omani populations include eczematous dermatitis, generalized pruritus, maculopapular rash (51), and urticarial rash (51), mostly after the first dose of vaccines (51). The aforementioned side effects are divided into local site injection reactions, new onset rash, and a flare-up of pre-existing condition (51). One woman in the Al Salmi et al., study reported itchy cherry angioma-like eruptions, which is a rarely described side effect (51). In line with these findings, a study from India reported five cases of healthcare workers who developed similar eruptive pseu-doangiomatosis after vaccination with the Astrazeneca vaccine (65). Erythromelalgia with skin peeling involving hands and feet, keratolysis exfoliative, excessive hair shedding, aphthous ulcers, dry lips with angular cheilitis, and nonspecific scaly erythematous plaques were also reported as uncommon/rare vaccine side effects (51). Moreover, another study in Oman reported the development of extensive deep vein thrombosis and pulmonary thromboembolism following vaccination with Pfizer-BioNTech first dose in 59-year-old women (30).

After vaccination, a condition called vaccine-induced immune-thrombotic thrombocytopenia (VITT) can occur due to an immune response reaction. This can lead to thromboembolism, which is associated with a variant of heparin-induced thrombocytopenia (HIT) (66). VITT usually presents as a combination of thrombocytopenia and venous and/or arterial thrombosis. It is linked to the presence of platelet factor 4-polyanion complexes (PF4) antibodies. PF4, through linking to the Fc receptor, induces a massive platelet activation which usually results in heparin-induced thrombocytopenia (HIT) (67). However, unlike the cases of heparin-induced thrombocytopenia, patients who were vaccinated did not receive heparin and thus the exact mechanism behind HIT development is still currently unclear (66). For instance, many VITT cases have been reported in several countries such as in Germany and Austria, following the uptake of the ChAdOx1 nCoV-19 vaccine (66). Other studies from Italy and Iran, for example, have linked VITT to BNT162b2 and Sinopharm vaccines, respectively (68, 69).

Similarly, vaccine-induced immune thrombosis and thrombocytopenia were also reported following the Pfizer-BioNTech vaccine after the second dose in Qatar along with other common local side effects such as pain, swelling, and erythema (29). Systemic side effects, on the other hand, including febrile reaction, muscle pain, and fatigue were also observed after the second dose of the Pfizer-BioNTech (29). Moreover, Razok et al, reported the development of Guillain-Barr´e syndrome following the same Pfizer-BioNTech receipt (29), which is also immune-mediated and encompasses a variety of demyelinating conditions such as acute inflammatory demyelinating polyradiculoneuropathy (AIDP) (70). This rare side effect was firstly reported from elderly women in the United States after receiving the Pfizer vaccine (71).

In the UAE, two studies have been conducted on the SinoPharm vaccine and showed that pain at the injection site, fatigue, and headache were the most commonly reported side effects after the second dose (15, 72). Indeed, it has been found that women experienced these side effects more frequently than men after the first dose of Sinopharm vaccine (15, 72). Side effects are more likely to be reported by previously infected individuals after the Pfizer-BioNTech vaccination (72). Studies in the literature have suggested that the administration of inactivated-virus vaccines, such as influenza, the attenuated Japanese encephalitis, and the attenuated Dengue vaccines, might result in a higher incidence of side effects in women. This suggests that women may develop a stronger immune response compared to males (73, 74). Moreover, in the same study, it was found that symptoms such as severe pain at the injection site, nausea, and muscle pain were more frequent in subjects younger than 49 years old after the first dose of SinoPharm and fatigue after the second dose (15). This is contrary to what has been declared by the Centers for Disease Control and Prevention, who stated that post-vaccination side effects are generally more intense after the first dose (73–75).

Increased reports of pain at the injection site, fatigue, headache, drowsiness, chills, muscle/joints pain, and fever side effects after the first dose of COVID-19 vaccines were also reported from three studies in the Jordanian population (76–78). Indeed, a significant association was found between the first dose of all covid-19 vaccines and a higher frequency of several side effects including chills, nausea, dizziness, sleepiness, laziness, abnormality in blood pressure, limb tingling, and numbness (77). Most of these side effects were moderate to severe, with the severity being linked to Astrazeneca, Pfizer-BioNTech, and Sinopharm vaccines (77) and the higher frequency being mainly attributed to Pfizer-BioNTech (76). In their paper, Nassar et al., observed that severity of side effects was generally at a higher level after the second dose of Pfizer-BioNTech, Sinopharm, Astrazeneca, and Sputnik V vaccines (78). Side effects that were commonly reported after the second dose included headaches and myalgias for Pfizer-BioNTech and tremor for Sinopharm (76). Some individuals even experienced thrombocytopenia and thrombosis after receiving the second dose of either Pfizer-BioNTech or Astrazeneca vaccines (77). The European Medicines Agency stated that there is an association between thrombocytopenia, thrombosis, and the Astrazeneca vaccine (79). Other side effects reported in the Jordanian population included bone and muscle pain after the receipt of the first dose of Astrazeneca, Pfizer-BioNTech, and Sinopharm vaccines. Side effects such as headache and cardiac events were found to be associated with female gende**r** after receiving both doses of Astrazeneca as well as the Pfizer-BioNTech vaccines (80). Indeed, headache was declared to be the most common side effect of Pfizer-BioNTech vaccine in a real-time analysis study of phase 1/2/3 and phase 3 clinical trials conducted in the United States, Germany, south Africa, Brazil, Argentina, and Turkey (81–83). Another study reported lower respiratory tract infection, lymphadenopathy, vasculitis, anxiety disorders, Guillain-Barre syndrome, and myopericarditis as uncommon side effects in the Jordanian population (84). Overall, vaccine side effects were found to be lower among individuals who received inactivated vaccines compared to other types such as the mRNA ones, in this case, the Sinopharm vaccine (80). This is in line with findings from the Chinese population, that vaccination side effects were significantly lower among individuals who received inactivated vaccines (85). Nevertheless, certain side effects were found to be associated with the first dose of Astrazeneca vaccine, including bone and muscle pain, flu-like symptoms, cardiac symptoms, psychological symptoms, dizziness, and gastrointestinal symptoms (80). It is worth mentioning that one study in Jordan noted that the elderly, those who use non-steroidal anti-inflammatory drugs (NSAIDs) on a regular basis, and individuals with allergies or rheumatoid arthritis were at a higher risk of experiencing COVID-19 vaccine side effects (78).

In Iran, fatigue, pain at the injection site, fever, and headache were the most commonly reported side effects after receiving the COVID-19 vaccines Astrazeneca, Sputnik V, and Sinopharm. Individuals with comorbidities were at higher risk of experiencing more side effects (86). The Astrazeneca vaccine was associated with more side effects than other vaccines (87, 88); this is thought to be related to the fact that it is a non-reproducible adenovirus carrier vaccine that uses a protein like the one produced by the SARS-CoV2 virus following a natural infection (89). It is worth mentioning that, in the Iranian population, hypertension was associated with a higher possibility of experiencing local side effects, while cardiac and cancer diseases were associated with increased odds of systemic side effects, all after the first dose of the vaccines. Allergy was the only comorbidity to be associated with higher odds of local as well as systemic side effects after the first dose of COVID-19 vaccines. On the other hand, cancer was the only comorbidity to be associated with mostly systemic side effects after the second dose of COVID-19 vaccines (88). Another study in Iran conducted on cancer patients found that the only side effect of Astrazeneca and Sputnik vaccines associated with cancer patients was mild fever (90). Other reported side effects included pain at the injection site, abdominal and body pain, headache, dizziness, shortness of breath, myalgia, chills, diarrhea, runny nose, and dry throat. Yet, none of these side effects needed special intervention (90). Unlike Sputnik and Sinopharm, no relation between gender and/or vaccine dose and Astrazeneca vaccine was found in the Iranian population. Womenreported more side effects than men following vaccination with Sinopharm (86, 88). Women reported more side effects after the first dose of Sputnik while men reported more after the second dose of it (86). Enayatrad et al., reported that the Barekat vaccine together with Sinopharm were associated with the fewest local as well as systemic side effects. Specifically, for Barekat, systemic side effects were lower after the first dose compared to the Sinopharm vaccine (88). The lower frequency of side effects observed after the Barekat vaccine could be due to its inactivated nature (88). Indeed, other studies have also linked inactivated vaccines with a lower frequency of side effects such as the Covishield vaccine (91). Moreover, in Iran, two cases of pemphigus vulgaris following the Astrazeneca vaccine have been described (92).

In Saudi Arabia, it has been reported that localized side effects including pain and swelling at the injection site, as well as systemic side effects such as fever, headache, and muscle pain were the most commonly reported after vaccination with Astrazeneca and Pfizer-BioNTech vaccines (93–97) (**Table 2**). Astrazeneca was linked to more side effects such as fatigue and headache than Pfizer-BioNTech (93, 98, 101, 102). This finding is consistent with a study conducted in Poland where more side effects were observed after vaccinations with Astrazeneca (118). Another study in Canada showed that, compared to other vaccine types, mRNA-based vaccines have fewer side effects (119). It is worth mentioning that, in KSA, uncommon vaccine side effects were reported; these included lower limb weakness, lymphadenopathy hypersensitivity, fast heartbeat, body rash, allergy, shortness of breath, flu-like symptoms, burning sensation in the eye (33), nausea (99), hypothyroidism, hypertension, anorexia, dry mouth, periodontitis, herpes zoster, low oxygen saturation, body malodor, pityriasis rosea, taste and smell distortion, left arm paresthesia, bleeding tendency, and thrombosis (106). This is in addition to seizure, loss of consciousness, high blood pressure, and hypoglycemia (99) (**Table 2**). Cutaneous side effects including angioedema have been also reported following vaccination with Pfizer-BioNTech and Astrazeneca in Saudi Arabia. This is with a significant association between these side effects and the subject’s pre-existing comorbidities (105). The most commonly observed comorbidities were diabetes, hypertension, and asthma. Furthermore, patients under treatment from antibiotics, antidepressants, or antihistamine drugs also experienced more cutaneous side effects following the receipt of these vaccines (105). Abnormalities in the menstrual cycle including increased duration and/or pain and hemorrhage were also uncommon side effects that corelated with the Pfizer-BioNTech, Astrazeneca (8, 96, 106), and SinoPharm vaccines (106). These observations are in line with studies from the United Kingdom and MENA Region (120, 121). Possible causes for the excessive menstrual bleeding could be platelet disorders, thrombocytopenia, and hormonal disruption (8, 122). Other uncommon side effects observed in the Saudi population includes hypersensitivity, fast heartbeat, and flu-like symptoms, with these being reported more frequently after the second dose of Pfizer-BioNTech (33). Flu-like symptoms could be caused by the immune response following vaccination, which consequently leads to cytokine production, causing an inflammatory effect on blood vessels, muscles, and other tissues (33). Other symptoms that were reported in Saudi Arabia after the administration of the Pfizer-BioNTech vaccine included arm and shoulder pain and swelling and redness at the injection site after the first dose (33). Some neurological side effects including impaired concentration, insomnia, and dizziness were also reported, with these being more frequently observed after the Astrazeneca vaccine. Chest pain and breathlessness also correlated to this same vaccine (8). Other reported rare neurological side effects include tinnitus, ear pain, numbness, tiredness, depression, anxiety, and depressive symptoms (123). As for the relation of previous COVID-19 infection with vaccine side effects, one study has shown that individuals with no previous infection experienced more side effects (102), especially after the second dose of Pfizer-BioNTech vaccine (35). In contrast, Mallhi et al, reported a significant association between previous Covid-19 infections and higher incidence of side effects after the first dose of Astrazeneca and the second dose of Pfizer-BioNTech vaccines (107). Another study found that previously infected individuals suffered more breathing difficulties compared to those without previous infection (33). In accordance with this study, studies in the United Kingdom and Iraq revealed that individuals previously infected with COVID-19 are at a higher risk of experiencing more side effects (109, 124). Previously infected individuals seem to exhibit higher antibody levels compared to those who have not been previously infected (109). Furthermore, a study in the United States implied that antibody response after vaccination in non-infected individuals is usually observed after the second dose of Pfizer-BioNTech and after the first dose in previously infected individuals (125). As for correlation of gender and/or age with vaccine side effects in Saudi Arabia, it has been found that fatigue and headache are more frequently observed in men and in individuals aged between 41-51 years old. This association could be due to the increased restlessness these individuals experience on account of senescence, along with their fear of receiving appropriate healthcare (95). In contrast, other studies in the KSA reported that young individuals (100–102, 104) women females (99, 101, 102, 104, 107) are prone to more side effects following vaccination. Interestingly, in their study Mallhi et al., and Al-Matouq et al., found that individuals with underlying comorbidities, specifically diabetes mellitus, hypertension (103, 107), hyperlipidemia (107), and asthma (103), experience more severe side effects after the receipt of Astrazeneca (107) and Pfizer-BioNTech vaccines (103). Nevertheless, having more than two comorbidities correlated with more side effects after both Astrazeneca and Pfizer-BioNTech vaccines (107). Comorbidities also correlated with an increased risk of side effects after the booster dose of Pfizer-BioNTech (103). Similarly, a large study in the UAE reported that individuals with comorbidities such as diabetes, hypertension, cardiovascular diseases, cancer, chronic lung diseases, and autoimmune diseases experienced more side effects after Sinopharm and Pfizer-BioNTech vaccines compared to those who did not have comorbid conditions (72). Other studies in the KSA, however, have reported that the presence of comorbidities including cardiovascular diseases, chronic obstructed pulmonary diseases (104), diabetes, hypertension (104, 106), anemia, allergy, and thyroid disorders (106) did not affect the incidence of side effects after COVID-19 vaccination (104, 106). In the literature, the correlation of pre-existing comorbidities with incidence and severity of COVID-19 vaccines has been controversial. While several studies confirmed this hypothesis (126, 127), others have rejected it (72, 128). A lack of consensus between studies could be due to the number and age of participants (127).

**Table 2:** COVID-19 Vaccine side effects in the general population of the Middle East.

Country	Vaccine type	Administered doses	Major common side effects	Minor less common side effects	Rare side effects	Reference
**KSA**	Pfizer & Astra	1 or 2 doses	N/V, joint/bone pain	Fatigue, redness & PIS, H/F, chills		(93)
	Pfizer & Astra	1 or 2 doses	Redness at injection site, seizures	H/F, fatigue, MJP		(98)
	Pfizer	2 doses	Dizziness, headache, N/V, fatigue	Swelling/rash at injection site, hoarseness, cough, chest pain	Acute transient loss of consciousness, high BP, hypotension, seizure, hypoglycemia	(99)
	Astra	1 dose	PIS, musculoskeletal symptoms, skin rash, fever, GI	PAL, chest pain, SOB, neurological		(100)
	Pfizer	At least 1 dose	Arm/shoulder pain, R/S at injection site, hyper- sensitivity symptoms, headache, flu-like symptoms	SOB, body rash, severe body allergy, eye burning sensation, bone/joint/back pain, fever, chills	Bell’s palsy	(33)
	Pfizer & Astra	1 or 2 doses	PIS, fatigue, muscle/abdominal/chest pain, headache, Insomnia, PAL, breathlessness, diarrhea, allergy	Abnormal menstrual cycle, sore throat, dry mouth	Anxiety, depression, sleepiness, mood disturbance, face numbness, leg bruises, DVT, HN, eye/ear/testis pain, blurred vision, tinnitus	(8)
	Pfizer & Astra	1 or 2 doses	General pain, fatigue, swelling, SOB	Headaches, dizziness, menstrual cycle changes, joint/abdominal pain, shivering, PAL		(101)
	Pfizer	1 or 2 doses	R/S & PIS, fatigue, MJP, H/F, chills, nausea	Lower limb weakness & loss of sensation, lymphadenopathy		(35)
	Pfizer & Astra	1 or 2 doses	Muscle pain, H/F, PIS with touch	Nausea, diarrhea, axillary lymphadenopathy		(102)
	Astra	At least 1 dose	PIS, myalgia, H/F, fatigue	Dizziness, weakness		(95)
	Pfizer & Astra	1 dose	PIS, fatigue, fever, myalgia, joint pain, malaise	Skin rash, cough, abdominal/joint pain, tachycardia, Syncope & numbness, blurred vision, cough, chills		(94)
	Pfizer	1 or 2 doses	Redness & PIS, H/F, fatigue, body pain			(103)
	Pfizer, Astra	1 or 2 doses	Fatigue, H/F, myalgia,			(104)
	Pfizer, Astra	1 or 2 doses	Swelling & redness & PIS	Rash, urticaria, angioedema		(105)
	Pfizer, Astra, Moderna	1 or 2 doses	RS & PIS, anxiety, dizziness, H/F, hoarseness, itchiness	Loss of consciousness, nausea, heartburn, sleep disruption, fatigue, seizures, anaphylaxis, SOB, wheezing, lips/face/throat swelling, ICU admission		(97)
	Pfizer, Astra, Sinopharm	At least 2 doses	Fatigue, myalgia, arthralgia, headache, dizziness		Anorexia, dry mouth, periodontitis, body malodor, herpes zoster, HN, hypothyroidism, low oxygen saturation, pityriasis rosea, taste & smell distortion, thrombosis, bleeding tendency & left arm paresthesia	(106)
	Pfizer	Booster dose	PIS, myalgia, H/F, fatigue			(96)
	Pfizer, Astra	2 doses	PIS, fatigue, H/F, MJP,			(107)
**Oman**	Pfizer	1 dose			Extensive DVT, pulmonary thromboembolism	(30)
	Pfizer & Astra	1 or 2 doses	PIS, tenderness at injection site, fever, body ache			(12)
**Bahrain**	Pfizer, Sputnik V, Astra, Sinopharm	2 doses	Fatigue, PIS, F/H, myalgia			(108)
**Iraq**	Pfizer, Astra, Sinopharm	Not specified	Fatigue, R/S & PIS, H/F, myalgia, muscle pain, chills, GI	Cough, SOB, LOS & taste, diarrhea		(109)
**Iran**	Sputnik V, Astra, Sinopharm	1 or 2 doses	Fatigue, chills, fever, skeletal/muscular pain	Diarrhea, sleepiness, loss of appetite, chest/abdominal pain, dyspnea, severe neurological effects		(86)
	Sputnik V, Sinopharm, Astra, Barekat	2 doses	Redness & PIS, fatigue, itching, bruising			(88)
	Sputnik V, Sinopharm, Astra, Barekat	2 doses	PIS, fatigue	Severe allergy, abdominal/chest pain, PAL, cardiac arrhythmia, high fever & agitation, vertigo, faint, high BP		(87)
	Sputnik V, Sinopharm, Astra	1 or 2 doses	H/F, PIS, dizziness, body & abdominal pain, chills, myalgia, SOB, diarrhea, runny nose, throat dryness			(90)
	Astra	One dose			Mucosal pemphigus vulgaris	(92)
**Qatar**	Pfizer	2 doses	Pain, swelling, erythema, febrile reaction, muscle pain, fatigue		Guillain–Barr´e syndrome	(29)
**UAE**	Sinopharm	1 or 2 doses	PIS, nausea, muscle pain, fatigue, headache, lethargy			(15)
	Sputnik V, Sinopharm, Astra, Pfizer	1 or 2 doses	PIS, fatigue, drowsiness, headache, MJP, R/S & enlargement of lymph nodes, itchiness, flu-like symptoms			(72)
**Egypt**	Pfizer, Astra, Sinopharm	1 or 2 doses	R/S & PIS, MJP, fatigue, lethargy, dizziness, H/F	Appetite decrease, N/V, diarrhea, cough, allergy, skin rash, runny nose		(110)
**Syria**	Sputnik V, Sinopharm, Astra, Pfizer, Sinovac, Moderna	1 or 2 doses	Tiredness, fatigue, H/F, chills, RS & PIS		Blood clots, thrombocytopenia, anaphylaxis, seizures, cardiac infarction	(111)
**Jordan**	Sinopharm & Pfizer	2 doses	PIS, fatigue, H/F, muscle pain, arthralgia, rigors	Skin rash, diarrhea, allergy, nausea		(76)
	Sinopharm, Astra, Pfizer, Sputnik V, Moderna, Covaxin, J & J	1 or 2 doses	Fatigue, swelling & PIS, H/F, sleepiness, chills, myalgia, joints pain	Nausea, abdominal/chest pain, GI, body bruises, bleeding, chills, itching, allergy, numbness, limbs tingling, dizziness, clogged/runny nose, dyspnea, IHB, abnormal BP, sore/dry throat, cough		(77b)
	Not specified	At least 1 dose	Sore arm at injection site, fatigue, MJP, drowsiness, headache	Anorexia, runny nose, sore throat, cough, eye pain, loss/change in the sense of taste/smell, lymphadenopathy		(78)
	Pfizer, Astra, Sinopharm	1 or 2 doses	R/S & PIS, fatigue, bone/muscles pain, H/F, chills	Cough, runny nose, LOS & taste, sore throat, depression, SOB, PAL, chest pain, loss of consciousness, paleness, bleeding		(80)
	Sputnik V, Sinopharm, Astra, Pfizer	1 or 2 doses	Fatigue, H/F, PIS	Lymphadenopathy, anxiety disorders, lower respiratory tract infection,	Guillain-Barre syndrome, vasculitis, myopericarditis	(84)
**Israel**	Pfizer	1 or 2 doses			Myocarditis	(112)
	Pfizer	1 or 2 doses			Myocarditis, HZV infection, thromboembolic, bell’s palsy	(113)
	Pfizer	1 or 2 doses			Myocarditis	(114
	Pfizer	1 or 2 doses			Myocarditis	(115)
	Pfizer	1 or 2 doses	Fatigue, H/F, myalgia, PIS, facial paranesthesia, chills, dizziness, arthralgia, nausea, diarrhea	Vomiting, cough, eye irritation, hoarseness, rash, facial swelling		(116)
	Pfizer	Booster dose			Myocarditis	(117)

Astra, AstraZeneca; J & J, Johnson & Johnson; SOB, shortness of breath; DVT, deep vein thrombosis; GI, gastrointestinal symptoms; BP, blood pressure; N/V, nausea/vomiting; LOS, loss of smell; HN, hypertension; R/S, redness and swelling; PAL, palpitation; IHB, irregular heartbeats; MJP, muscle & joint pain; ICU, intensive care unit.

In Syria, individuals with comorbidities, in particular diabetes mellitus, reported more frequent mild side effects compared to subjects without (111). On the other hand, pre-existing underlying conditions such as respiratory and hematological disease and allergies were associated with the frequency of more severe side effects (111). This is in line with other studies conducted in the USA and Mexico where patients who were at a greatest risk of developing side effects post-vaccination include those with a history of type-2 diabetes (129, 130). The USA study also linked hypertension, hyperlipidemia, allergies, and kidney and heart diseases with more severe side effects after the receipt of mRNA-based vaccines (130). Indeed, in this country (Syria) the most common reported side effects were pain at the injection site, fatigue, muscle pain, headache, and fever (111). Tiredness and fatigue were mostly observed after vaccination with Astrazeneca and Sputnick, headache was mostly observed with Moderna, and low- and high-grade fever were mostly observed with Johnson & Johnson and Astrazeneca, respectively (111). Pain at the injection site being the most frequent side effect could be due to what is called ‘Covid arm’, which is a delayed but innocuous injection site allergic reaction that usually resolves without treatment (131). The intensity of side effects after vaccination in Syria varied depending on the type of vaccine (111). Astrazeneca vaccination was generally associated with severe side effects, while Sputnik was associated with mild side effects, and Johnson & Johnson vaccine with moderate side effects (111). These side effects, especially the severe ones, were reported more frequently after the second dose of most vaccines, including Astrazeneca, Pfizer-BioNTech, Sinopharm, and Moderna (111).

In Bahrain, pain at the injection site, which is the most frequent side effect observed in almost all middle eastern countries, was most highly reported after the first dose of Pfizer-BioNTech vaccine, followed by Astrazeneca, and Sinopharm. Fatigue was also observed following the first dose of COVID-19 vaccines (108). Pizer-BioNTech and Sputnik were highly linked to frequent reports of fever and headache after the first dose. Nausea was observed in individuals after receiving the first and second doses of Pfizer-BioNTech vaccine but only after the first dose of Astrazeneca (108).

In Iraq, pain at the injection site was reported more frequently after Pfizer-BioNTech compared to Astrazeneca and Sinopharm vaccines. Women reported significantly more adverse effects than men. Iraqi women reported more side effects than men especially after both doses of the Pizer-BioNTech vaccine (47). Most of the other common side effects included fatigue, headache, fever, chills, and myalgia; these were mostly associated with the Astrazeneca vaccine. Astrazeneca was also linked to higher severity of side effects and to those aged less than 50 years old (109). This finding contrasts with the statement of the Food and Drug Administration (FDA) who stated that individuals aged 55 years or more are less likely to experience adverse effects (75). Similarly, randomized controlled trials conducted in Brazil, South Africa, and the UK on the safety and efficacy of the Astrazeneca vaccine revealed that side effects tend to be less intense and less reported in older individuals (132). Presence of comorbidities including hypertension, diabetes, thyrotoxicosis, and asthma, as well as a history of COVID-19 infection, were found to increase the risk of more frequent side effects after vaccination with Pfizer-BioNTech, Astrazeneca, and Sinopharm vaccines (109).

In Egypt, pain at the injection site, fatigue, muscle pain, and fever were found to be the most frequent side effects observed after receiving the first dose of Pfizer-BioNTech, Astrazeneca, and Sinopharm. Specifically, side effects were more frequently reported after Astrazeneca receipt (110). Other rarely observed side effects reported from the Egyptian population included decreased appetite, inflammation of the nervous system, loss of sensation, convulsions, tremors, numbness, loss of sensation, and tingling (110). All of these side effects were more severe after the first dose of Sinopharm and Astrazeneca vaccines and after the second dose of Pfizer-BioNTech.

In Israel, all vaccine side effects were reported more frequently after the second dose of Pfizer-BioNTech in young individuals. Facial paranesthesia, a rarely observed side effect, was reported in one study after the first as well as the second dose of this same vaccine (116). Indeed, studies in Israel have shown that rare side effects were noticed increasingly after the vaccination with Pfizer-BioNTech. Rare side effects included thromboembolic events, myocarditis (112–115, 117), as well as Bell’s palsy, appendicitis, and Herpes zoster infection (113). For Bell’s palsy, in their study, Ozonoff et al., suggested a possible link between this side effect and the receipt of Pfizer-BioNTech (133). This linkage is in contrast to what the FDA have stated: that no significant association exists between Bell’s palsy and COVID-19 vaccines (134). Indeed, the increase in the incidence of Herpes zoster infections following vaccination with Pfizer-BioNTech could explain the increase of Bell’s palsy cases, due to the association between Herpes zoster infection and Bell’s palsy cases. Herpes zoster infection is one of the potential causes of Bell’s palsy (135, 136). The severity of the reported rare side effects was often mild, with some of them being described as potentially serious such as myocarditis. In most of the cases, myocarditis symptoms appeared after the second dose of Pfizer–BioNTech vaccine (112–114) with the incidence being higher in men aged less than 30 years (112, 114) (113). This finding is in contrast to the phase 3 clinical trials of Pfizer–BioNTech vaccine conducted in the United States, Germany, South Africa, Brazil, Argentina, and Turkey (83). This controversy could be due to the small sample size reported in Israel studies compared to the clinical trials (112, 114) (113). Myocarditis symptoms were also reported in one study after the booster dose of the Pfizer–BioNTech vaccine (117). These side effects were less observed after the booster dose compared to the second dose of the Pfizer–BioNTech vaccine (117). Moreover, in their study, David et al., found that pregnant women experienced fewer side effects than controls who were matched by age and ethnicity. Pregnant women were less likely to report vaccine side effects, including fatigue, myalgia, headache, chills, and fever (116). Possible reasons for this finding could be either due to different pharmacokinetics that result in the masking of vaccine side effects by pregnancy symptoms or the minimal expectation of this group following vaccination (137). Another reason could be due to the reluctance of pregnant women to expose their fetus to any possible harm, thereby suppressing their feeling of side effects (138).

## High-risk population

According to the Centers for Disease Control and Prevention, individuals tend are more likely to experience severe vaccine side effects as their number of underlying clinical comorbidities increases (75, 139). Furthermore, the presence of underlying comorbidities increases the likelihood of hospitalization as well as death from COVID-19; these comorbidities include but not limited to diabetes, respiratory disease, chronic heart disease, chronic kidney disease (CKD), chronic liver disease, neurological disease, and conditions requiring immunosuppressive medication. Individuals suffering from medical conditions are prioritized for vaccination in many countries (140–142). The effectiveness of COVID-19 vaccinations in the general population appears to be the subject of numerous clinical trials; data from the real world has been used to support these clinical trials. However, for those in the high-risk group, the effectiveness and safety of COVID-19 vaccination has not yet been estimated.

### Sickle cell anemia patients

In Oman, in patients with sickle cell anemia, thromboembolic complications, fever, a significant decline in hemoglobin and platelets, and an increase in liver enzymes, particularly alkaline phosphatase, were reported following the receipt of the Astrazeneca vaccine (50). Furthermore, these patients experienced vaso-occlusive crisis and lethal TTP-like illness with thromboembolic consequences. It is unclear if the fact that all patients had S/B0 thalassemia was a factor in the emergence of these complications (50). Most vaccine-induced immune-thrombotic thrombocytopenia (VITT) cases were linked mostly to two adenovirus vector COVID-19 vaccines, namely Astrazeneca and Janssen, with this event being reported more frequently after Astrazeneca (143). Interaction between the vaccine and the platelets or PF4 is a possible pathway of pathogenesis (66). Moreover, the Astrazeneca vaccine could induce immune-medicated antibody response or the generation of antibodies against platelet PF4, which may lead to major activation of platelets causing this vaccine-induced immune-thrombotic thrombocytopenia (50, 66). In their study, Greinacher et al. and Nazy et al. stated that antibody reaction could also lead to severe pain along with a vaso-occlusive crisis in patients with sickle cell anemia (66, 144).

### Autoimmune inflammatory rheumatic patients

In Israel, Furer et al., studied the efficacy and safety of the Pfizer-BioNTech vaccine among autoimmune inflammatory rheumatic (AIIRD) patients and found that this vaccine was immunogenic in most patients. The seropositivity rate that the antibody immune response was induced by vaccination with was lower in AIIRD patients than in healthy vaccinated controls (49). AIRD patients reported similar side effects to those in heathy controls. Major side effects were also reported in AIIRD patients including non-disseminated Herpes zoster, uveitis, and pericarditis (49). The incidence of Herpes zoster was not specifically associated with AIIRD patients or Pfizer-BioNTech in clinical trials (145). Indeed, several Herpes zoster cases were reported after vaccination with Pfizer-BioNTech in healthy individuals who did not receive an immunosuppressive treatment in other studies (146, 147).

### Highly allergic patients

In Israel, most severely allergic individuals who received the COVID-19 vaccines did not experience any immediate side effects. Rather, these patients experienced anaphylaxis mostly after the first dose and an allergic reaction after both the first and second doses of Pfizer-BioNTech vaccine (48). Acute or late-onset allergic reactions could be caused either by the released IgE antibodies in response to a vaccine component or through additional possible mechanisms that lead to rapid mast cell activation (48). Rare immediate allergic reactions following the first dose of Pfizer-BioNTech were also reported in some cases, including swelling of the tongue or uvula and skin flushing. Anaphylactic reactions were also reported, involving bronchospasm, shortness of breath, skin eruption, angioedema, and gastrointestinal symptoms (48) (**Table 1**). Immediate anaphylactic reactions and their appearance after vaccination with BNT162b2 were revealed by the CDC (148). Some common non-allergic side effects were also reported in allergic individuals including dizziness, fatigue, headache, muscle pain, paresthesia, and vagal reactions. After the second dose of Pfizer-BioNTech, only flushing and cough were reported (48).

### Multiple sclerosis patients

Two Iranian studies found that there is no association between vaccination with Sinopharm and specific side effects in patients with multiple sclerosis (52, 53). However, some patients experienced neurological side effects including motor and vertigo symptoms (53). The incidence of the neurological side effects was associated with the presence of comorbidities, receipt of natalizumab therapy, as well as post-vaccination myalgia (53).

Other reported side effects following vaccination with Astrazeneca and Sinopharm in Multiple sclerosis Iranian patients include injection site pain, tenderness, redness, fever, headache, fatigue, nausea, diarrhea, and muscle pain (54). Side effects were more commonly observed after vaccination with the Astrazeneca vaccine compared to Sinopharm (54). Furthermore, it has been reported in these patients that systemic side effects were more frequently observed after the first dose of Sinopharm vaccine compared to the second dose (53).

## Children and adolescents

In children and adolescents, Pfizer–BioNTech and Moderna vaccines were approved for use by the end of December 2021 and by June 2022, respectively (149). The CDC recommended COVID-19 vaccines for every individual aged 6 months and older, and boosters for those aged 5 years and more (150). To date, there have been no large-scale studies that have explored and provided safety profiles of COVID-19 vaccines among children and adolescents, especially in middle eastern countries.

In Saudi Arabia, most adolescents and children who were vaccinated with Pfizer-BioNTech vaccine reported at least one side effect. More individuals were admitted to hospitals and took medication after the second dose than the first. The need to visit doctors and hospitalizations due to vaccine side effects in this category could be due to the parents’ concerns about their children (55). Mild to moderate pain at the injection site, fever, tiredness (55, 56), nausea, vomiting, headache, chest pain, and shortness of breath (55) were reported by Saudi adolescents and children (**Table 1**). In parallel, the Centers for Disease Control and Prevention and a meta-analysis by Du et al., on the safety and efficacy of COVID-19 vaccination in children and adolescents revealed that the most often reported side effects were pain at the injection site, minor headaches, fatigue, and chest pain (75, 151). More frequent side effects were associated with previous SARS-CoV-2 infection (55). Immunity might lead to higher side effects after the second dose in previously infected children, implying the immunogenicity and effectiveness of the vaccine (55, 124).

In Israel, Guy Witberg et al., found that, following Pfizer-BioNTech receipt, common side effects together with a mild course of myocarditis were recorded for Israeli adolescents aged 12 to 16 years. Perimyocarditis and myocarditis events were detected only in men after the second dose of Pfizer-BioNTech (58, 59). Myocarditis could be considered an initial presentation of COVID-19 infection in rare cases, as part of multisystem inflammatory syndrome (MIS-C) in children and adolescents (152). Snapiri et al. reported that several vaccinated subjects without prior COVID-19 infection also experienced myocarditis as a side effect (58). Indeed, in this later study, none of the patients with myocarditis exhibited any clinical or laboratory signs consistent with MIS-C such as fever, rash, conjunctivitis, or hemodynamic abnormalities, nor elevated levels of inflammatory biomarkers (58). Worldwide, there are just a few cases of perimyocarditis and myocarditis reported in adolescents after Pfizer-BioNTech vaccination. Perimyocarditis was previously reported as a side effect for several other vaccines, including human papillomavirus, hepatitis A, and Influenza vaccine in individuals aged 18 years or less (58, 153).

In Iran, Soberana and Sinopharm vaccines were the most common vaccines administered to children (57). The most commonly reported side effects in children aged less than 18 years old were general common side effects such as fatigue, pain at the injection site, and fever. More severe side effects were also reported including ataxia, arrhythmia, pericarditis, and seizures (57).

## Conclusion

This review summarized the current knowledge on the most common, less common, and rare COVID-19 vaccines side effects in the middle eastern population. Pain at the injection site followed by fatigue, fever, and headache were the most common side effects reported. No conclusive result on the efficacy and/or adverse event of each vaccine can be drawn for this population. Overlapping adverse events between different COVID-19 vaccines as well as between different countries has been observed. The severity and frequency of side effects were the points of contention. Differences in these factors could be attributed to different study populations, vaccine platforms, and evaluation methods. Indeed, it only appears that the female gender, lower age, previous SARS-COV-2 infection, and presence of underlying comorbidities are the major factors that predispose subjects to a higher frequency of vaccines side effects. Nevertheless, this could be not conclusive for all middle eastern countries. This is because data on this issue are scarce or even missing in several countries including Lebanon, Yemen, Syria, Kuwait, and Bahrain. Future research should cover missing data on this area. Furthermore, more research should be conducted regarding the long-term side effects, side effects after booster doses, and side effect differences in the case of heterologous and homologous vaccination. This would allow for a better understanding of the COVID-19 vaccines’ impact and enable proper handling of the patients who experience these side effects.

## Author contributions

WA: Conceptualization, Funding acquisition, Project administration, Resources, Supervision, Visualization, Writing – review & editing. GM: Conceptualization, Data curation, Methodology, Writing – original draft. ID: Conceptualization, Methodology, Validation, Visualization, Writing – review & editing.
